# Dynamic changes in intestinal microbiota in young forest musk deer during weaning

**DOI:** 10.7717/peerj.8923

**Published:** 2020-04-13

**Authors:** Yimeng Li, Minghui Shi, Tianxiang Zhang, Xin Hu, Baofeng Zhang, Shanghua Xu, Jianhong Ding, Defu Hu, Shuqiang Liu

**Affiliations:** 1 School of Ecology and Nature Conservation, Beijing Forestry University, Beijing, China; 2 Beijing Key Laboratory of Captive Wildlife Technology, Beijing Zoo, Beijing, China

**Keywords:** Forest musk deer, Weaning transition, Intestinal microbiota

## Abstract

Weaning is an important event for all mammals, including young forest musk deer. However, weaning stress may cause intestinal microbiota-related disorders. Therefore, high-throughput 16S rRNA gene sequencing was applied to study the dynamic changes in intestinal microbiota during pre-weaning (10 days before weaning) and post-weaning (10 days after weaning) in 15 young forest musk deer. We saw that intestinal microbiota diversity in the post-weaning period was significantly higher than that in the pre-weaning period. The most dominant bacterial phyla were similar in the two groups (*Firmicutes*, *Bacteroidetes* and *Verrucomicrobia*). Meanwhile, we applied Linear discriminant analysis effect size (LefSe) to identify the most differentially microbial taxa in the pre-weaning and post-weaning groups. In the post-weaning forest musk deer, the relative abundance of *Actinobacteria*, *Spirochaetes*, *Ruminococcaceae_UCG-005*, *Treponema* and *Prevotella* was higher than in the pre-weaning group. However, higher relative abundance of the phyla Bacteroidetes was found in the pre-weaning group compared with that in the post-weaning group. In summary, this research provides a theoretical foundation for the dynamics of young forest musk deer intestinal microbiota during the weaning transition, which may benefit in understanding the growth and health of forest musk deer.

## Introduction

Forest musk deer (*Moschus berezovskii*) are small ruminants unique to Asia, which belong to the family *Moschidae*, order Artiodactyla, Mammalia. The deer have the following biological characteristics: alertness, timidity, solitude, sensitivity and high stress-susceptibility. Forest musk deer are widely distributed in the mountainous forest area of Southwest China, mainly concentrated in Gansu, Shaanxi, Sichuan, Tibet provinces, etc. (Sheng & Liu, 2007). In the 1960s, there were more than 2.5 million wild musk deer in China. However, since the 1970s, the forest musk deer population in the wild has decreased dramatically due to excessive hunting, deforestation and habitat fragmentation (Yang et al., 2003). In 2002, forest musk deer was listed under Class I protected species in China and then considered endangered on the IUCN Red List (IUCN, 2004). Since 1958, captivity breeding of the forest musk deer was attempted in China to alleviate wild resource depletion, and musk deer farming in China is relatively successful compared to other countries (Shrestha, 1998). However, in captivity, the incidence of intestinal diseases and mortality rate of forest musk deer is high. According to an investigation, the mortality rate of forest musk deer in the 1st month after birth was approximately 25%, whereas that during weaning was nearly 35% (Qi, 2011).

For the young, weaning is a complex period which usually accompanied by physiological, psychological, nutritional, etc. changes (Waran, Clarke & Famworth, 2008). During weaning, baby forest musk deer are separated from their mother, depriving them of their mother’s care, her breast milk, which was easily digestible and a familiar environment, leading them to a completely independent life. According to Hu et al. (2013) research, in terms of behavior, the young forest musk deer showed intense and frequent restlessness in the first few days after weaning, most of them sniffing, tweeting, running, looking for mother musk deer, trying to cross the wall, etc.; in terms of food intake, within the first 5 days after weaning, the young forest musk deer took little food, even stopped eating, resulting in negative weight gain (the mean weight change was −1.1%). About 1 week after weaning, weight gain began to change from negative to positive. Therefore, in such cases, according to our laboratory’s another research (Wang et al., 2016), the psychological and physiological state of the young forest musk deer is disturbed, which is also reflected by a sharp rise in cortisol levels. Such weaning stress is also common in other mammals such as foals and piglets (Bruschetta et al., 2017; Li et al., 2018). Hence, this important event may easily cause intestinal microbiota related-disorders, such as diarrhea (Smith et al., 2010).

The intestinal microbiota plays a crucial role in host health. The microbial community has numerous roles including aiding colonic fermentation, stimulating the immune system and protection from pathogenic bacteria (Tremaroli & Bäckhed, 2012; Martin et al., 2010; Buffie & Pamer, 2013), and its alteration has already been linked with numerous diseases or infections (Rinninella et al., 2019). Based on previous research, the host’s gastrointestinal reacts to stress hormones through synthesizing neurotransmitters and cytokines (Holzer & Farzi, 2014), which might modify microbiota composition and influence the overall health and performance of hosts.

To improve the health conditions of forest musk deer, an in-depth understanding of its intestinal microbiota is essential. Although several studies have been performed on the intestinal microbiota in adult forest musk deer (Li et al., 2017; Hu et al., 2017), there is a dearth in understanding the dynamics of intestinal microbiota in young forest musk deer during weaning. Therefore, this study aimed to describe the shift in the fecal bacterial community in healthy young forest musk deer before and after weaning using the 16S rRNA gene high throughput sequencing technology, which will benefit the growth, health and management of captive forest musk deer.

## Materials and Methods

### Ethics statement

This study was carried out in accordance with the recommendations of the Institution of Animal Care and the Ethics Committee of Beijing Forestry University. The protocol was approved by the Ethics Committee of Beijing Forestry University. The collection of fecal samples was approved by the Jiuyao Forest Musk Deer Breeding Center.

### Sample collection

In this study, 15 young forest musk deer (six males, nine females; vaginal delivery) having the similar time of birth and similar weights were selected from the Qingchuan Jiuyao forest musk deer breeding center. All the young forest musk deer presents no obvious illness and behavioral abnormalities during sampling. Young forest musk deer were weaned at 80 days of age, separated from the mother musk deer and transferred to a new enclosure. Fecal samples at pre-weaning (70 days of age) and post-weaning (90 days of age) were collected during August–September, 2018. Due to the intense reaction of young forest musk deer in the first few days after weaning, therefore, to avoid affecting young forest musk deer, we collected fecal samples 10 days before/after weaning for analyzing. Generally, the young forest musk deer only feeds on breast milk within 1 month after birth. After 1 month and before weaning, in addition to breast milk, they begin to eat a small amount of mulberry leaves (*Morus alba*) and concentrate (containing wheat bran, corn, soybean meal and salt). After weaning, the young musk deer fed entirely on mulberry leaves and concentrate. Water was offered *ad libitum*. None of the 15 young forest musk deer received anthelmintic or antibiotic treatments since their birth until the end of the study. Our sampling protocol was operated as follows: each enclosure was totally cleaned at night and then fresh fecal of each young forest musk deer was collected in the early morning next day. Enclosures are sterilized once a week, and we only collected part of fresh fecal samples that have not touched the ground when sampling. Our colleagues worn disposable sterile gloves to collect fecal samples and then put them into sterile centrifuge tubes to prevent contamination. The samples were kept in liquid nitrogen and transported to Beijing Forestry University laboratory then kept at −80 °C until DNA extraction.

### DNA extraction, 16S rRNA gene amplification and sequencing

We extracted bacterial DNA with the QIAamp DNA Stool Mini Kit (QIAGEN, Hilden, Germany) following the standard protocol. The DNA concentration and purity were measured by Qubit dsDNA HS Assay Kit (Life Technologies, Carlsbad, CA, USA). The V3–V4 hypervariable region of bacterial 16S rRNA gene was amplified from all the 30 fecal samples using primers 338F (5′-ACTCCTACGGGAGGCAGCA-3′) and 806R (5′-GGACTACHVGGGTWTCTAAT-3′). The polymerase chain reaction (PCR) volume was 50 μl containing 10 μl PCR buffer, 0.2 µL High-Fidelity DNA Polymerase, 1 µL dNTP, 10 µL GC Enhancer, 1.5 µL each of 10 µM forward and reverse primers, 60 ng template DNA and the rest volume was DNase-free sterile water. The PCR conditions were as follows: 95 °C for 5 min, followed by 25 cycles of 95 °C for 30 s, 50 °C for 30 s, 72 °C for 40 s and 72 °C for 7 min. The PCR products was purified with DNA gel extraction kit (Axygen, Shanghai, China). Ultimately, high-throughput sequencing on an Illumina HiSeq 2500 platform (Illumina, Inc., San Diego, CA, USA) was conducted at Biomarker Technologies Corporation (Beijing, China).

### Bioinformatic analysis

The program PRINSEQ (Schmieder & Edwards, 2011) was used for quality filtration. Reads with length <200 bp, or contained homopolymers >8 bp, were discarded. And UCHIME was applied for chimera checking. All Sequences were grouped into operational taxonomic units (OTUs) with a 97% sequence similarity level using the UCLUST program (v1.2.22) (Edgar, 2010) against the SILVA database (Pruesse et al., 2007). Alpha diversity indices (observed OTUs and Shannon), which were calculated in Mothur software (Schloss et al., 2009), indicate bacterial community richness and diversity. Non-metric multidimensional scaling (NMDS) based on the unweighted UniFrac distance matrices was performed to determine beta diversity using the ggplot2 in R (Version 3.5.2). One-way analysis of similarity (ANOSIM) was used to test the differences in bacterial communities between pre-weaning and post-weaning groups using the vegan package in R. Linear discriminant analysis effect size (LefSe) (https://huttenhower.sph.harvard.edu/galaxy/) was used to identify statistically significant taxa in pre-weaning and post-weaning group (Segata et al., 2011). All raw sequences obtained during this study were submitted to Figshare (https://doi.org/10.6084/m9.figshare.11553513.v1).

## Results

### Sequence statistics

**Figure 1:**
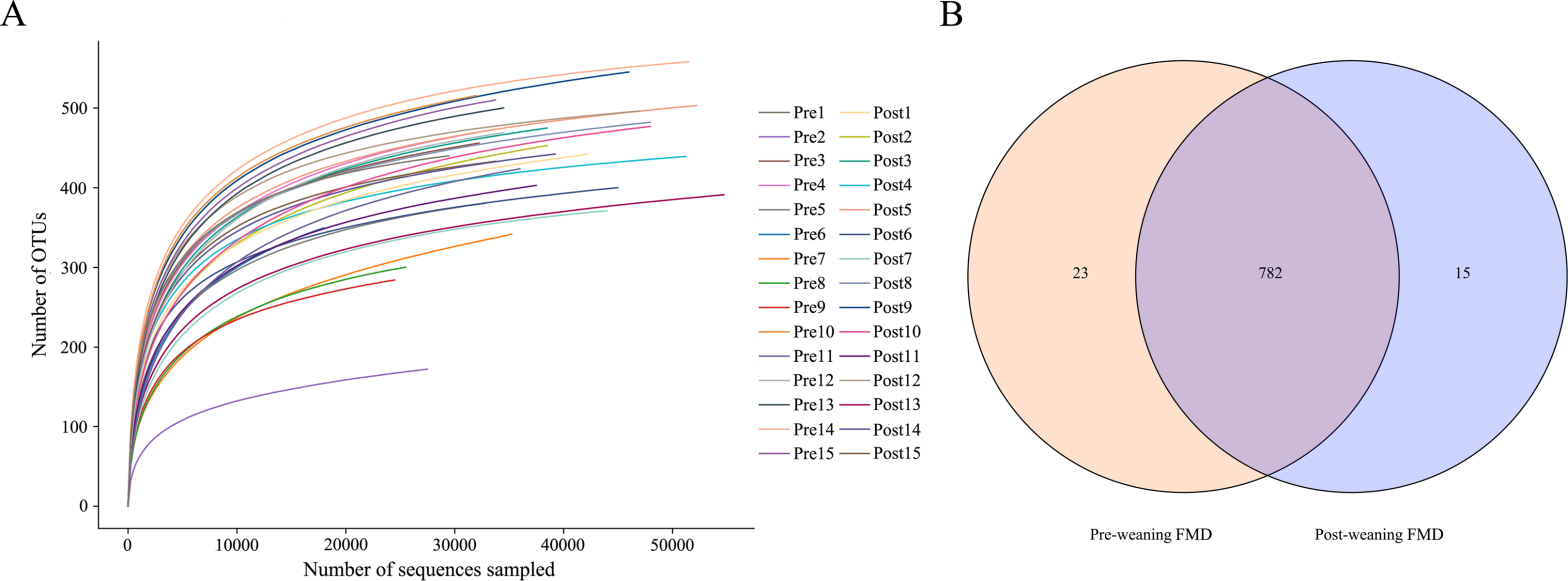
Rarefaction curves and Venn diagram. (A) The rarefaction curves of OTUs. The *x*-axis shows the number of valid sequences per sample and the *y*-axis shows the observed species (operational taxonomic units, OTUs). Each curve in the graph represents a different sample and is shown in a different color. As the sequencing depth increased, the number of OTUs also increased. Eventually the curves began to plateau, indicating that as the number of extracted sequences increased, the number of OTUs detected was decreased. (B) The Venn diagrams show the numbers of OTUs (97% sequence identity) that were shared or not shared by pre-weaning and post-weaning individuals, respectively, depending of overlaps (FMD, forest musk deer).

A total of 1,525,616 effective sequences were obtained from 30 fecal samples of 15 young forest musk deer and 24,564–71,287 (mean 50,854 ± 11,218) effective sequences (mean length = 413.4 bp) were obtained from each fecal sample. A total of 820 OTUs were identified at 97% sequence similarity level and these OTUs were classified into 15 phyla, 22 classes, 36 orders, 61 families and 154 genera. Rarefaction curves demonstrated that almost all the bacterial species were detected in feces of young forest musk deer (Fig. 1A). There were 782 core OTUs in the bacterial communities and 23 genera were found in the pre-weaning forest musk deer only, while 15 genera were detected solely in the post-weaning forest musk deer (Fig. 1B).

### Alpha diversity and beta diversity analysis of pre-weaning and post-weaning forest musk deer

**Figure 2:**
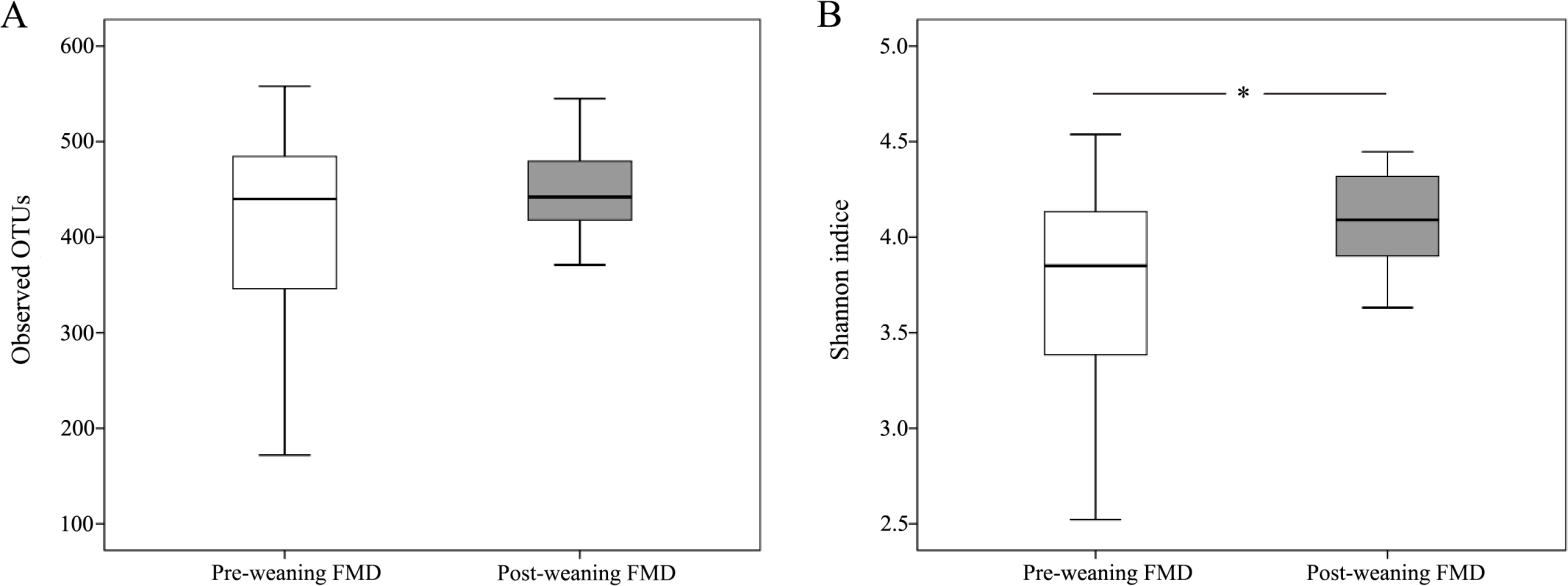
Variations in alpha diversity of the pre-weaning and post-weaning forest musk deer. (A) Comparisons of the number of observed OTUs between pre-weaning and post-weaning forest musk deer. (B) Comparisons of Shannon indices between pre-weaning and post-weaning forest musk deer. In all panels, boxes represent the interquartile range (IQR) between the first and third quartiles. The lines inside boxes represent the median. Whiskers denote the lowest and highest values within 1.5 IQR from the first and third quartiles, respectively. *P < 0.05 (Student’s *t*-test) (FMD, forest musk deer).

To further investigate the dynamic changes in intestinal microbiota during the weaning period, we calculated the alpha and beta diversity of young forest musk deer intestinal microbiota. Alpha diversity was evaluated according to the observed OTUs and the Shannon indices (Fig. 2). The observed OTUs, depend on species richness, showed no significant difference between the pre-weaning (411.33 ± 105.27) and post-weaning (450.13 ± 47.29) young forest musk deer (*P* = 0.208). The Shannon index, depend on both species richness and evenness showed significant differences between the pre-weaning (3.76 ± 0.57) and post-weaning (4.08 ± 0.26) groups (*P* = 0.036).

**Figure 3:**
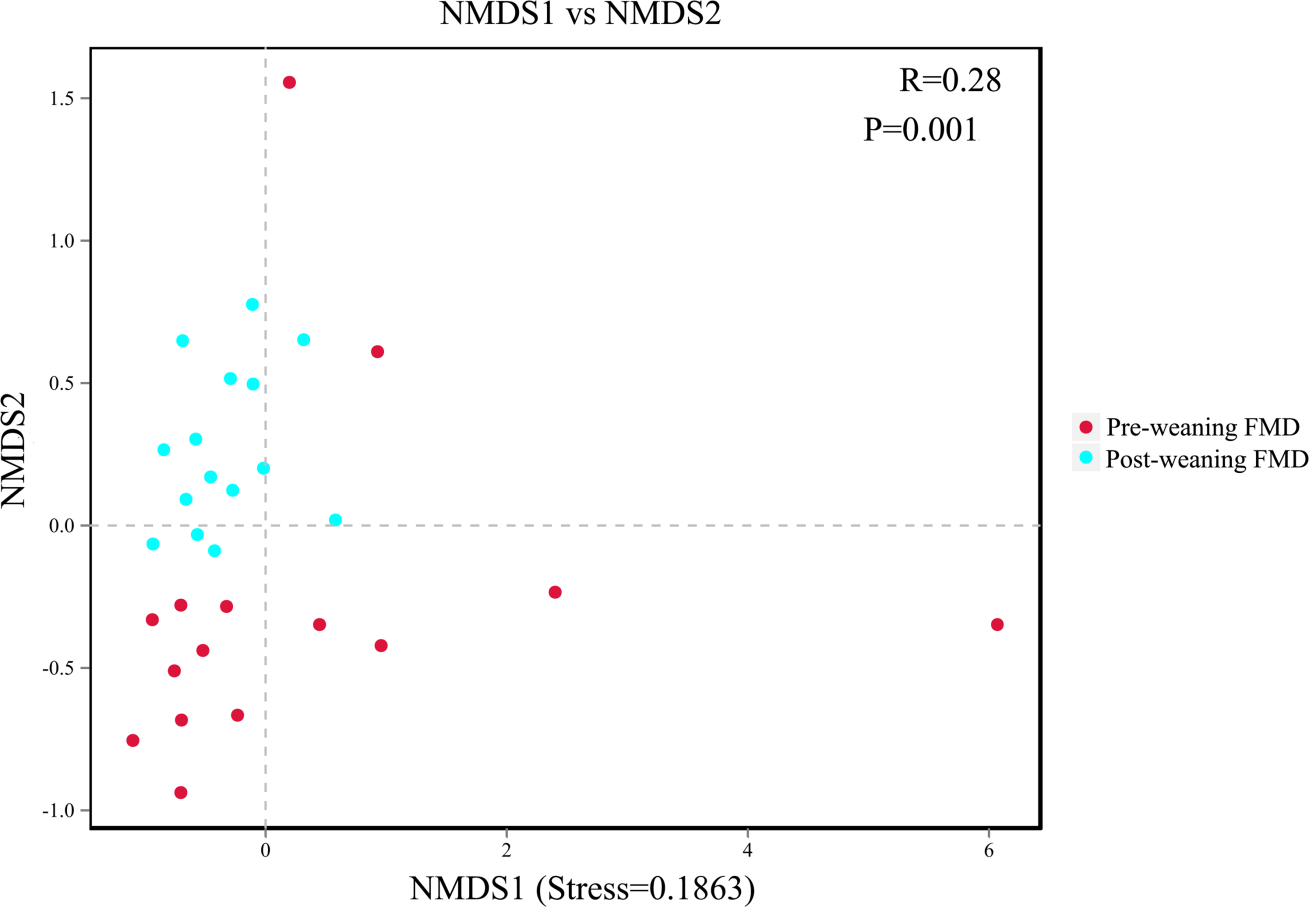
NMDS analysis. Each point represents one sample, and different colors represent different groups. The distance between points represents the level of differences; stress lower than 0.2 indicates that the NMDS analysis is reliable. The greater distance between two points infers a higher dissimilarity between them (FMD, forest musk deer).

Beta diversity was used to determine whether there was a difference in bacterial community compositions between pre-weaning and post-weaning groups. The NMDS based on unweighted UniFrac distance revealed that the intestinal microbiota of young forest musk deer showed obvious segregation from pre-weaning to post-weaning (ANOSIM: *R* = 0.28, *P* = 0.001) (Fig. 3), which indicated a shift in the intestinal bacterial community of pre-weaning and post-weaning young forest musk deer.

### Differences in microbial community composition in pre-weaning and post-weaning forest musk deer

**Figure 4:**
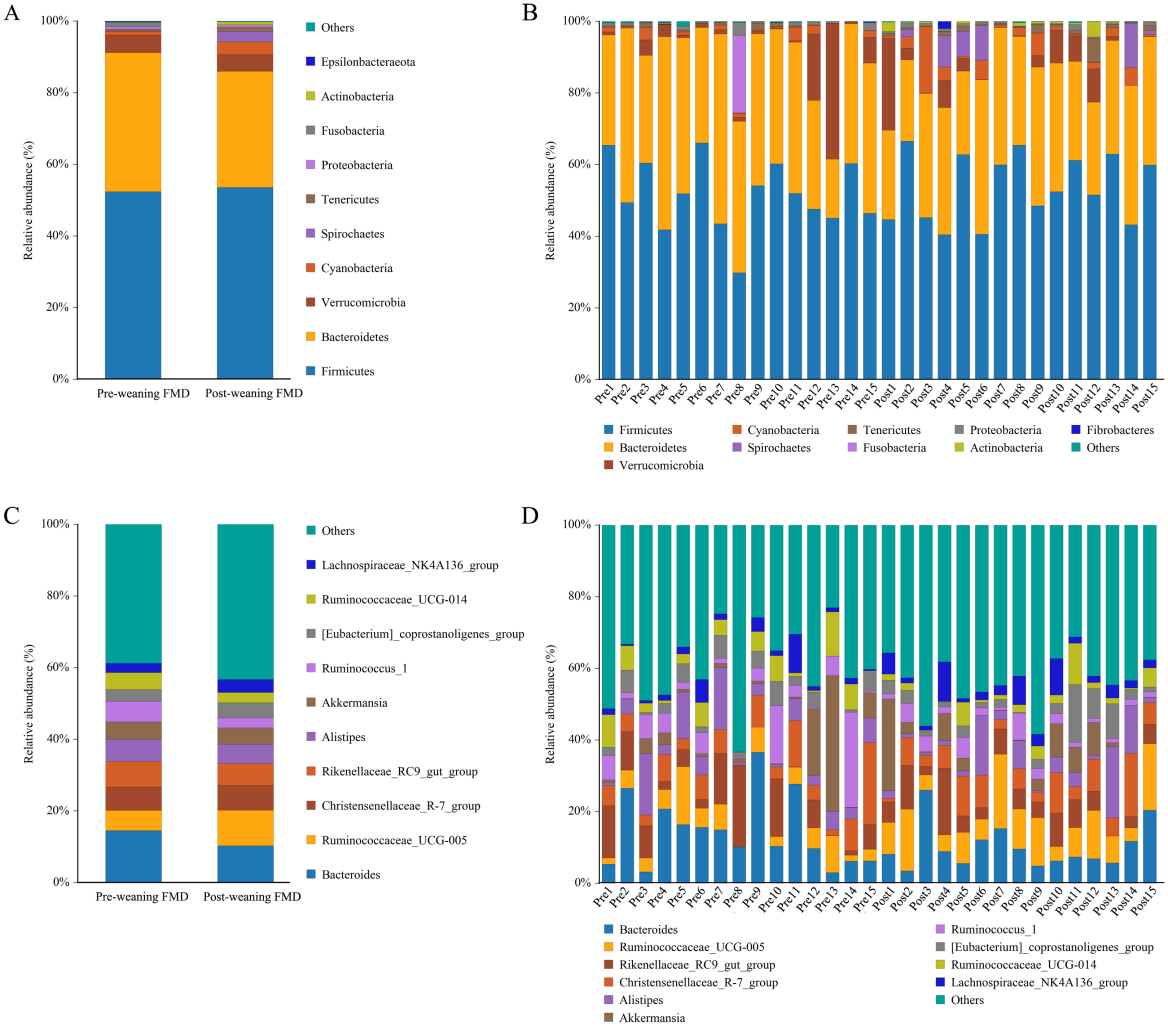
Bar chart of relative abundance. Relative abundance (%) of the ten most abundant bacteria phyla ((A) for groups, (B) for individuals) and genera ((C) for groups, (D) for individuals) obtained from 30 fecal samples of forest musk deer in pre-weaning and post-weaning. Less than 1% abundance of the phyla or genus was merged into others (FMD, forest musk deer).

The relative abundance of the top 10 abundant bacterial phyla and genera of intestinal microbiota in pre-weaning and post-weaning forest musk deer is shown in Fig. 4. The top 10 phyla in the two groups were *Firmicutes* (52.35% in pre-weaning and 53.59% in post-weaning), *Bacteroidetes* (38.85% in pre-weaning and 32.41% in post-weaning), *Verrucomicrobia* (4.86% in pre-weaning and 4.63% in post-weaning), *Cyanobacteria* (1.13% in pre-weaning and 3.61% in post-weaning), *Spirochaetes* (0.02% in pre-weaning and 2.83% in post-weaning), *Tenericutes* (0.49% in pre-weaning and 1.24% in post-weaning), *Proteobacteria* (0.74% in pre-weaning and 0.65% in post-weaning), *Fusobacteria* (1.24% in pre-weaning and 0.02% in post-weaning), *Actinobacteria* (0.07% in pre-weaning and 0.81% in post-weaning) and *Epsilonbacteraeota* (0.10% in pre-weaning and 0.03% in post-weaning).

The top 10 genera in the two groups were *Bacteroides* (14.55% in pre-weaning and 10.26% in post-weaning), *Ruminococcaceae_UCG-005* (5.58% in pre-weaning and 9.98% in post-weaning), *Christensenellaceae_R-7_group* (6.53% in pre-weaning and 6.93% in post-weaning), *Rikenellaceae_RC9_gut_group* (7.13% in pre-weaning and 6.11% in post-weaning), *Alistipes* (6.22% in pre-weaning and 5.34% in post-weaning), *Akkermansia* (4.85% in pre-weaning and 4.63% in post-weaning), *Ruminococcus_1* (5.71% in pre-weaning and 2.71% in post-weaning), *Eubacterium* (coprostanoligenes_group) (3.31% in pre-weaning and 4.29% in post-weaning), *Ruminococcaceae_UCG-014* (4.73% in pre-weaning and 2.86% in post-weaning) and *Lachnospiraceae_NK4A136_group* (2.55% in pre-weaning and 3.60% in post-weaning).

**Figure 5:**
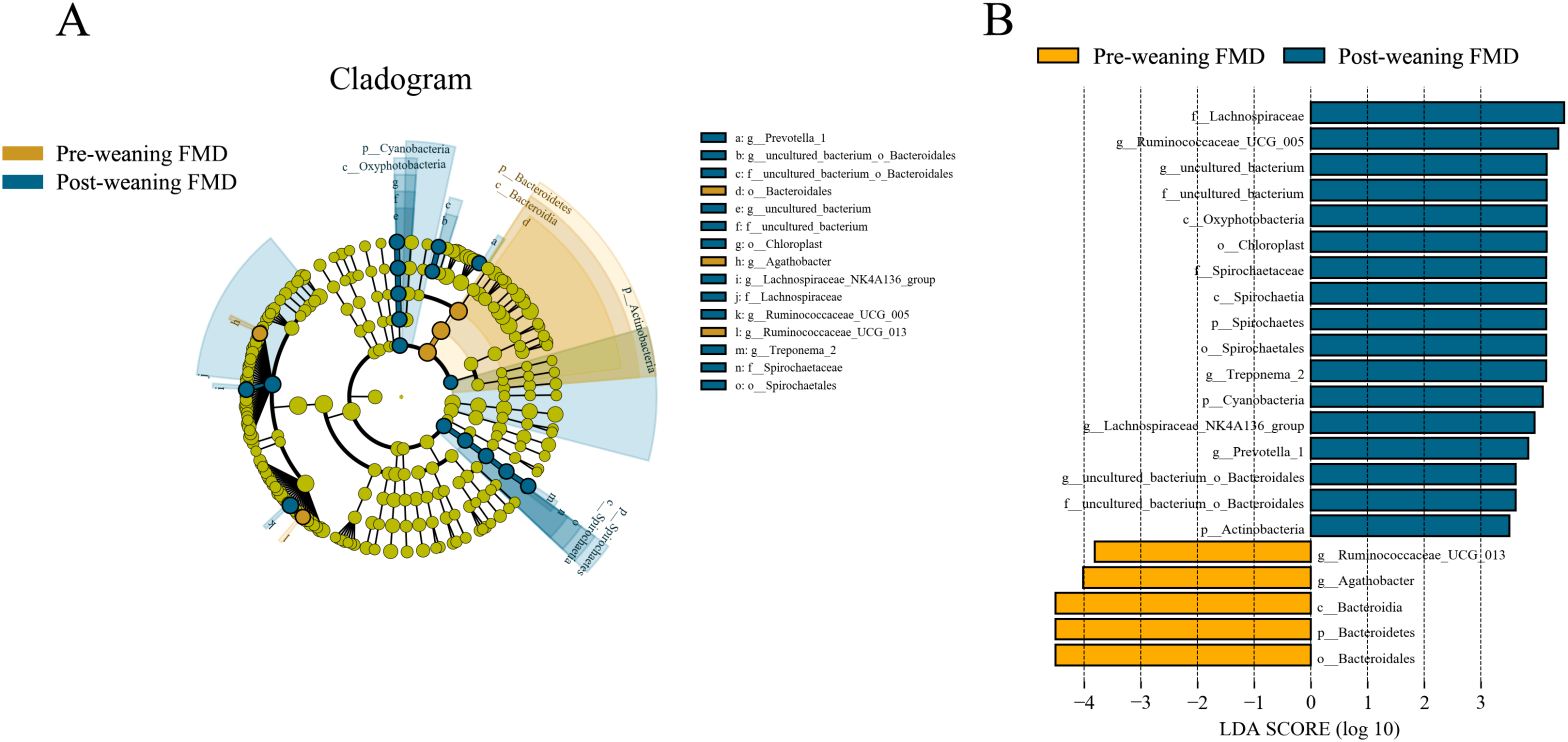
Differently abundant taxa identified using LEfSe analysis. (A) A cladogram showing the differences in relative abundance of taxa at five levels between pre-weaning and post-weaning. The plot was generated using the online LEfSe project. The orange and blue circles mean that pre-weaning and post-weaning showed differences in relative abundance and yellow circles mean non-significant differences. (B) Plot from LEfSe analysis. The plot was generated using the online LEfSe project. The length of the bar column represents the LDA score. The figure shows the microbial taxa with significant differences between the pre-weaning (orange) and post-weaning (blue) (LDA score > 3.5) (FMD, forest musk deer).

To study the differences in intestinal microbiota between pre-weaning and post-weaning forest musk deer, Lefse was performed. In post-weaning forest musk deer, at the phylum and genus level, the relative abundance of *Actinobacteria*, *Spirochaetes*, *Ruminococcaceae_UCG-005 Treponema* and *Prevotella* was higher than in the pre-weaning forest musk deer. whereas, the abundance of *Bacteroidetes* was higher in the pre-weaning forest musk deer compared to the post-weaning forest musk deer (Fig. 5).

## Discussion

Weaning is the most important event in the early life of mammals. During this process, young forest musk deer lose their mother’s care and their food intake shifts from breast milk to plants and concentrate. Moreover, their lifestyle changes to them becoming completely independent, leading to stress, which could in turn affect their health by causing intestinal microbiota disorders. Our study investigated the dynamic changes in intestinal microbiota in pre-weaning and post-weaning young forest musk deer.

In terms of alpha diversity, the observed OTUs (richness) showed no difference between the pre-weaning and post-weaning groups while the Shannon index (diversity) in post-weaning deer was significantly higher than that in pre-weaning deer. Based on a theory, high species diversity offers “functional redundancy”, which helps to maintain the stability and resistance of ecosystem under environmental pressure (Konopka, 2009). Therefore, high bacterial diversity is usually believed beneficial for host health (Turnbaugh et al., 2009; Le Chatelier et al., 2013) and it is also regarded as a sign of matured intestinal microbiota. In terms of intestinal microbiota composition, NMDS and ANOSIM analysis demonstrated that there were significant differences in intestinal microbiota composition between pre-weaning and post-weaning young forest musk deer. Consistent with the previous studies on forest musk deer (Li et al., 2017), Firmicutes and Bacteroidetes were the two most dominant bacterial phyla in pre-weaning and post-weaning young forest musk deer. Similar results were also found in human infants (Bäckhed et al., 2015), piglets (Kim et al., 2012) and foals (Mach et al., 2017), suggesting similarities in gut bacterial compositions between young forest musk deer and other mammals. At the genus level, *Bacteroides* and *Ruminococcaceae_UCG-005* were the two most dominant bacterial genera in young forest musk deer intestinal microbiota. From pre-weaning to post weaning, the abundance of *Bacteroides* decreased while the abundance of *Ruminococcaceae_UCG-005* increased. Significant changes in the composition of intestinal microbiota during weaning were also observed, which is also common in other species, such as horses and pigs (Mach et al., 2017; Hu et al., 2016).

To further study the differences between pre-weaning and post-weaning forest musk deer, Lefse was performed which showed that the abundance of Bacteroidetes was significantly higher in pre-weaning forest musk deer compared to that in post-weaning forest musk deer. *Ruminococcaceae_UCG-005* and *Prevotella* was significantly higher in post-weaning than in pre-weaning young forest musk deer. After weaning, young musk deer no longer consume breast milk, but mainly feed on leaves and concentrate. Previous studies showed that Bacteroidetes have the capacity to degrade macromolecular organic matter, such as polysaccharides and proteins (Thomas et al., 2011). *Ruminococcaceae_UCG-005* belong to the phylum Firmicutes and are cellulolytic bacteria (Evans et al., 2011). *Prevotella* are highly active hemicellulolytic bacteria (Matsui et al., 2000). We believe that the high abundance of *Bacteroides* in the pre-weaning forest musk deer may be because of their capability to consume breast milk oligosaccharides, while the high abundance of *Prevotella* in the post-weaning forest musk deer may be attributed to their capability to degrade hemicelluloses exists in plants (Hayashi et al., 2007; Lamendella et al., 2011). The existence of these bacteria is thus important for non-cellulosic plant polysaccharide and to increase utilization efficiency in hosts (Kabel et al., 2011). It is worth mentioning that according to some previous studies, the relative abundance of bacteria in the genus *Bacteroides* decrease under the influence of stressors (Bailey et al., 2011; Dinan & Cryan, 2012). This may be another reason for the decrease in *Bacteroides* after weaning due to weaning stress; however, the underlying mechanism needs further evaluation.

## Conclusions

In conclusion, to date, information on forest musk deer intestinal bacteria dynamics during the weaning period was limited. This research provides basic information on the bacterial diversity and composition of healthy young forest musk deer undergoing the weaning transition. Due to the fact that diet is the single variable in our research, thus we speculate that the change in intestinal microbiota composition is influenced primarily by dietary differences between pre-weaning and post-weaning forest musk deer; however, weaning stress may also affect the composition of the intestinal microbiota. Therefore, in the future, the weaning process can be carried out step by step, separate young musk deer from mother musk deer at progressively increasing intervals every day, reducing the stress response caused by sudden separation. Further research in this field is required to better understand whether the alterations of intestinal microbiota during weaning period might affect the immunity level of the host. Altogether, this study will benefit the growth, health and management of captive forest musk deer.

## Additional Information and Declarations

### Data Availability

The following information was supplied regarding data availability: The raw sequences are available at GenBank (PRJNA609129) and Figshare: LI, YIMENG; Shi, Minghui; Zhang, Tianxiang; Zhang, Baofeng; Hu, Xin; Xu, Shanghua; et al. (2020): Raw data.zip. figshare. Dataset. DOI 10.6084/m9.figshare.11553513.v1.
